# Association of acrylamide exposure with markers of systemic inflammation and serum alpha-klotho concentrations in middle-late adulthood

**DOI:** 10.3389/fpubh.2025.1457630

**Published:** 2025-03-19

**Authors:** Lin Gan, Jiaoyang Wang, Kang Qu, Wei Jiang, Yuhong Lei, Ming Dong

**Affiliations:** ^1^Department of Neurology and Neuroscience Center, The First Hospital of Jilin University, Changchun, China; ^2^Cancer Institute, The First Hospital of Jilin University, Changchun, China

**Keywords:** acrylamide, glycidamide, systemic immune-inflammation index, system inflammation response index, *α*-klotho

## Abstract

**Background:**

Acrylamide (AA) is a ubiquitous environmental contaminant linked to systemic inflammation and oxidative stress in animal studies; however, the epidemiological evidence is still lacking. This study aimed to evaluate the association of AA exposure with markers of systemic inflammation and serum concentrations of an anti-aging protein, *α*-klotho.

**Methods:**

The study used data of 1,545 adults aged 40–79 years from the National Health and Nutrition Examination Survey (NHANES) 2013–2016. Internal AA exposure was assessed using hemoglobin adducts of acrylamide and glycidamide (HbAA and HbGA, respectively), the sum of HbAA and HbGA (HbAA + HbGA), and the ratio of HbGA and HbAA (HbGA/HbAA). Two novel indicators, systemic immune-inflammation index (SII) and system inflammation response index (SIRI), were calculated using the lymphocyte, platelet, neutrophil, and monocyte counts. The serum concentration of soluble *α*-klotho was measured using enzyme-linked immunosorbent assay. Multivariable linear regression models were used to estimate the associations of AA hemoglobin biomarkers with systemic inflammation indicators and serum concentration of *α*-klotho.

**Results:**

Each one-unit increase in ln-transformed HbAA, HbGA, and HbAA+HbGA was associated with an increase in SII in models adjusted for age, sex, and race/ethnicity [regression coefficient (*β*) = 32.16, 95% confidence interval (CI): 3.59, 60.73; β =36.37, 95% CI: 5.59, 67.15; and β = 37.17, 95% CI: 6.79, 67.55, respectively]. However, the associations were no longer significant after additional adjustment for lifestyle factors. Higher HbAA and HbAA+HbGA predicted lower serum *α*-klotho concentrations (*β* = −35.76 pg./mL, 95% CI: −63.27, −8.25; β = −33.82 pg./mL, 95% CI: −62.68, −4.96, respectively).

**Conclusion:**

The hemoglobin adducts of AA parameters, as biomarkers of internal AA exposure, were associated with reduced serum concentrations of *α*-klotho among the United States population in their middle-late adulthood. The findings indicated that exposure to AA may have impacts on the molecular pathways of aging and related diseases by influencing *α*-klotho concentrations.

## Graphical abstract

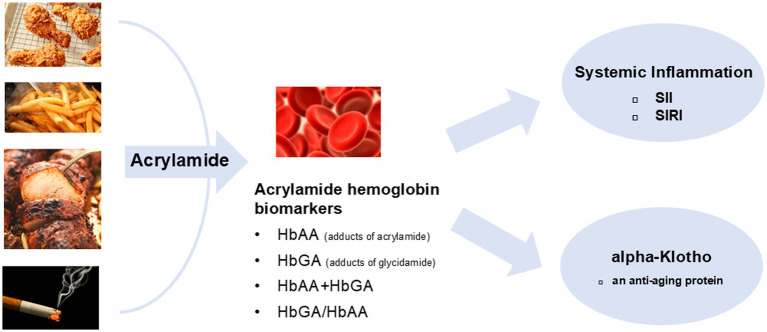

## 1 Introduction

Acrylamide (AA) is a reactant extensively used to synthesize polyacrylamide polymers, gels, and binding agents (1). AA has attracted public attention in the last decades because it can be developed via Maillard reaction during food processing at high temperatures, such as frying and baking (2). Meanwhile, it is also found in the smoke generated when tobacco burns in a lit cigarette (3). Thus, AA can be absorbed into the body through ingestion, inhalation, and dermal contact with AA-containing products (3, 4). Diet contributes to an average daily intake of 0.02–1.53 μg/(kg body weight · day) AA for the general population (5). Once absorbed, AA is widely distributed to various organs and metabolized to a major metabolite, glycidamide (GA), in the liver (6). Hemoglobin adducts of AA (HbAA) and GA (HbGA) are validated biomarkers in human biomonitoring and commonly found in the United States (US) population (7). The ubiquitous presence of AA has raised health concerns worldwide owing to its toxicological effects (1, 2).

AA exposure has been related to various adverse health outcomes, such as cancer (8, 9), cardiovascular diseases (10), respiratory diseases (11), diabetes (12), and depression (13, 14). It impacts human health through multiple mechanisms. Particularly, AA exposure increases systemic inflammation (2). *In vitro* and *in vivo* studies indicated that AA treatment activated the nuclear factor-κB (NF-κB) pathway and enhanced the release of pro-inflammatory cytokines (15, 16). However, evidence of AA exposure associated with systemic inflammation in humans is still scarce. Recently, two novel indicators derived from lymphocyte, neutrophil, monocyte, and platelet counts were introduced: the systemic immune-inflammation index (SII) and the system inflammation response index (SIRI) (17). Initially, SII was used to assess the prognosis of patients with liver cancer, whereas SIRI predicted survival after chemotherapy in patients with cancer (18, 19). These indicators were widely used for evaluating systemic inflammation response in the general population because of their easy access and biological significance (20, 21).

Another toxicological mechanism of AA-associated health outcomes is oxidative stress damage. AA or GA contributes to the depletion of glutathione, overproduction of reactive oxygen species (ROS), and alteration of the nuclear factor erythroid 2-related factor 2 pathway (22). Oxidative stress may be inhibited by soluble *α*-klotho, which is a transmembrane protein related to the aging process (23). α-klotho downregulates ROS-associated stress and prolongs cellular lifespan (24, 25). It also maintains the anti-aging process and prevents aging-related diseases. Therefore, exploring a potential link between AA exposure and soluble *α*-klotho may have significant public health implications.

AA exposure induces systemic inflammation and oxidative stress in animals (1); however, the epidemiological evidence related to this is quite limited. Previous epidemiological studies suggested that hemoglobin or urinary biomarkers of AA and GA were associated with increased levels of pro-inflammatory cytokines and inflammatory markers, including low-grade inflammation score (INFLA-score), C-reactive protein (CRP), circulating mean platelet volume (MPV), and high-sensitivity interleukin-6 (IL-6) (10, 26–28). However, few studies have addressed the associations of HbAA and HbGA with novel markers of systemic inflammation and serum concentrations of *α*-klotho. Therefore, this study aimed to explore the associations of AA exposure with markers of systemic inflammation and serum concentrations of α-klotho in general adults aged 40–79 years using the National Health and Nutrition Examination Survey (NHANES) 2013–2016 cycles.

## 2 Materials and methods

### 2.1 Study design and population

The study data were extracted from the NHANES database (29). NHANES is a population-based survey aiming to evaluate the health and nutrition of participants in the US. This nationally representative survey included physical examinations, laboratory tests, dietary information, and health-related questionnaires. The NHANES team captured informed consent from each participant *prior to* enrollment. The study protocol (Protocol #2011–17) was reviewed and approved by the NCHS Research Ethics Review Board.

**Figure 1:**
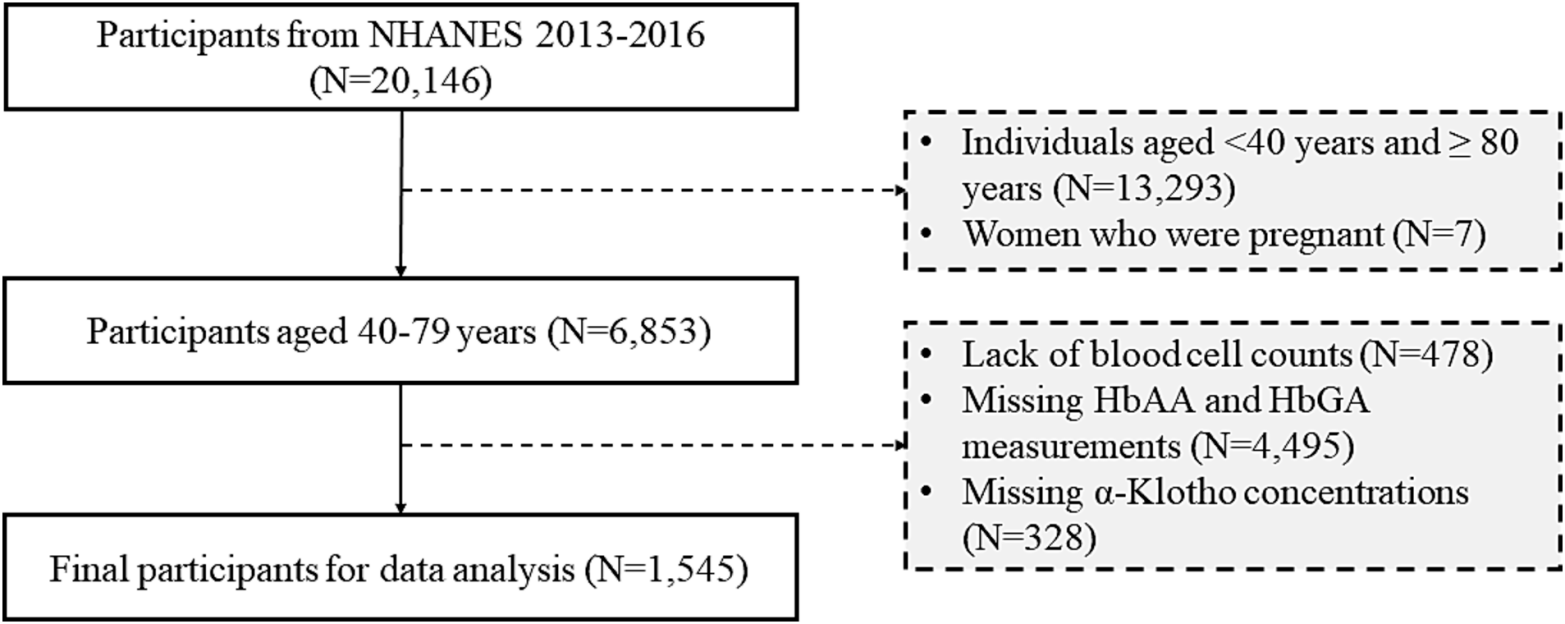
The number of participants included in the current analysis in NHANES survey 2013–2016.

The NHANES 2013–2014 and 2015–2016 cycles were selected owing to data availability. A total of 20,146 participants were enrolled in NHANES 2013–2016 cycles (Figure 1). Adults aged 40–79 years were included (*N* = 6,853). Pregnant women were excluded at the examination (*N* = 7). Furthermore, participants with missing blood cell counts (*N* = 478), missing HbAA and HbGA measurements (*N* = 4,495), and missing *α*-klotho concentrations in serum (*N* = 328) were excluded from the analysis.

### 2.2 Measurements of AA and GA concentrations

The concentrations of HbAA and HbGA in human whole blood or erythrocytes were measured (7) as described in a previous study (30). Briefly, the adducts of AA and GA were cleaved using a modified Edman reaction. The Edman products were prepared by liquid–liquid extraction and quantified using high-performance liquid chromatography–tandem mass spectrometry. The limit of detection of AA and GA was 3.90 pmol/g Hb and 4.90 pmol/g Hb, respectively. Laboratory quality assurance and quality control protocols are available on the NHANES website (29).

### 2.3 Systemic immune-inflammation index

The whole blood specimens were analyzed at NHANES mobile examination centers using automated hematology analyzing devices (29). After analysis in duplicate, the observed results were averaged to improve the data quality. We calculated SII using the counts of peripheral blood cells (1,000 cells/μL):


$$\frac{\mathrm{Platelet}\;\mathrm{count}\times \mathrm{neutrophil}\;\mathrm{count}}{\mathrm{Lymphocyte}\;\mathrm{count}}$$

SIRI was also calculated as follows (19):


$$\frac{\mathrm{Monocyte}\;\mathrm{count}\times \mathrm{neutrophil}\;\mathrm{count}}{\mathrm{Lymphocytes}\;\mathrm{count}}$$

### 2.4 Serum concentrations of *α*-klotho

Serum concentrations of soluble α-klotho from the participants were quantified using an extensively validated IBL enzyme-linked immunosorbent assay method (31). The sensitivity of the assay was 6 pg./mL. The samples were analyzed in duplicate to ensure the precision. The final values were calculated using the average of the two observed values.

### 2.5 Covariables

As reported in previous studies (10, 28), several potential confounding factors in relation to AA exposure and systemic inflammation/*α*-klotho were considered, including socio-demographic characteristics, physical examination, dietary information, and lifestyles. The covariables were included if they changed the coefficient of AA hemoglobin biomarkers by greater than 10% in simple linear regression models. The following covariables were selected: sex, age, race/ethnicity, educational level, family poverty-income ratio (PIR), body mass index (BMI), cigarette smoking (smoker or non-smoker), alcohol consumption (days per year) and physical activity (minutes per week). Cigarette smoking was assessed by individual’s self-report. Smoker was defined as participants who smoked at least 100 cigarettes in life. In the smoking subgroup analysis, an additional continuous variable of an average number of cigarettes smoked per day over the past 30 days was adjusted in the models for smokers. A continuous variable of alcohol consumption was generated from three components: consumption of at least 12 alcohol drinks/lifetime (yes or no), frequency of drinking alcohol over past 12 months (0 to 365) and days of alcohol consumption per week, month, or year. “0” was assigned to individuals who did not have at least 12 alcohol drinks/lifetime. The amount of alcohol consumption was calculated by day × frequency for those who had 12 or more alcohol drinks. Physical activity was calculated as the total weekly minutes of vigorous work activities, moderate work activities, walking or bicycling, vigorous recreational activities, and moderate recreational activities (32).

### 2.6 Statistical analysis

The general characteristics of participants were summarized using the median and interquartile range for continuous variables and frequency and proportion for categorical variables. HbAA, HbGA, HbAA + HbGA, and HbGA/HbAA were natural logarithm (ln) transformed owing to the skewed distribution of residuals. Spearman correlation coefficients were calculated to evaluate pairwise correlations of AA hemoglobin indicators. Multiple imputations with chained equations were applied for a few missing covariables, including family PIR, BMI, cigarette smoking, and alcohol consumption.

Multivariable linear regression models were used to explore the associations of AA hemoglobin biomarkers with SII, SIRI, and serum concentration of *α*-klotho. The collinearity of the linear regression models was assessed using a variance inflation factor, revealing no multi-collinearity. Regarding covariables, three models were used. Model 1 was a crude model without any adjustment. Model 2 was a basic model adjusted for sex, age, and race/ethnicity. Model 3 was adjusted for all the aforementioned covariables as the primary model. Generalized additive models with 3-degrees-of-freedom natural cubic splines were fitted to estimate the potential nonlinear associations of AA hemoglobin biomarkers with markers of systemic inflammation and serum concentrations of *α*-klotho. Tobacco smoke is a major source of AA exposure (33). Therefore, an interaction term between cigarette smoking and target biomarkers (data shown in Supplementary Table S1) was further introduced, and then stratified analysis based on cigarette smoking was performed.

Statistical analysis was performed using Stata version 17.0 (Stata Corp, TX, United States) and R version 4.2.1.^1^ Statistical significance was considered as a two-sided *p* < 0.05 and *p* < 0.10 for interaction terms.

## 3 Results

A total of 1,545 participants were included in the present analysis. The general characteristics and outcomes are presented in Table 1. The frequency and proportion of adults aged 40–59 years were 851 and 55.1%, respectively. A majority of participants were non-Hispanic White (39.7%) and had higher educational levels (54.0%).

**Table 1:** Descriptive statistics of general characteristics of 1,545 participants from NHANES 2013–2016.

Characteristics	N (%)	Variables	Median (25th-75th percentile)
Age (years)		BMI (kg/m^2^)	28.7 (24.9, 33.1)
40–59	851 (55.1)	PIR	2.20 (1.15, 4.39)
60–79	694 (44.9)	Endpoints	
Sex		SII	455 (332, 623)
Male	763 (49.4)	SIRI	1.07 (0.75, 1.58)
Female	782 (50.6)	α-klotho (pg/mL)	779 (649, 961)
Race/Ethnicity		Target analytes	
Mexican American	265 (17.2)	HbAA (pmol/g Hb)	41.5 (32.2, 60.6)
Other Hispanic	185 (12.0)	HbGA (pmol/g Hb)	36.2 (26.7, 51.0)
Non-Hispanic White	614 (39.7)	HbAA+HbGA (pmol/g Hb)	78.2 (60.1, 111.0)
Non-Hispanic Black	269 (17.4)	HbGA/HbAA	0.822 (0.689, 0.982)
Other race - including multi-racial	212 (13.7)		
Education			
Less than 9th grade	197 (12.8)		
9th–11th grade	181 (11.7)		
High school grade	333 (21.5)		
Some college	417 (27.0)		
College graduate or above	417 (27.0)		
Cigarette smoking			
Smoker	738 (47.8)		
Non-smoker	807 (52.2)		

N, frequency; %, proportion; BMI, body mass index; PIR, poverty income ratio; SII, systemic immune-inflammation index; SIRI, system inflammation response index.

HbAA and HbGA were detected in all the samples. The median values (25th percentile, 75th percentile) of HbAA, HbGA, and HbAA + HbGA were 41.5 (32.2, 60.6) pmol/g Hb and 36.2 (26.7, 51.0) pmol/g Hb and 78.2 (60.1, 111) pmol/g Hb, respectively. The median value of HbGA/HbAA was 0.822 (0.689, 0.982) (Table 1). HbAA and HbGA were highly correlated with a Spearman correlation coefficient of 0.822 (*p* < 0.001). The median (25th percentile, 75th percentile) SII and SIRI values were 455 (332, 623) and 1.07 (0.75, 1.58), respectively. A high correlation was observed between SII and SIRI (Spearman correlation coefficient: 0.747, *p* < 0.001). The median *α*-klotho concentration in serum was 779 (649, 961) pg./mL (Table 1).

**Figure 2:**
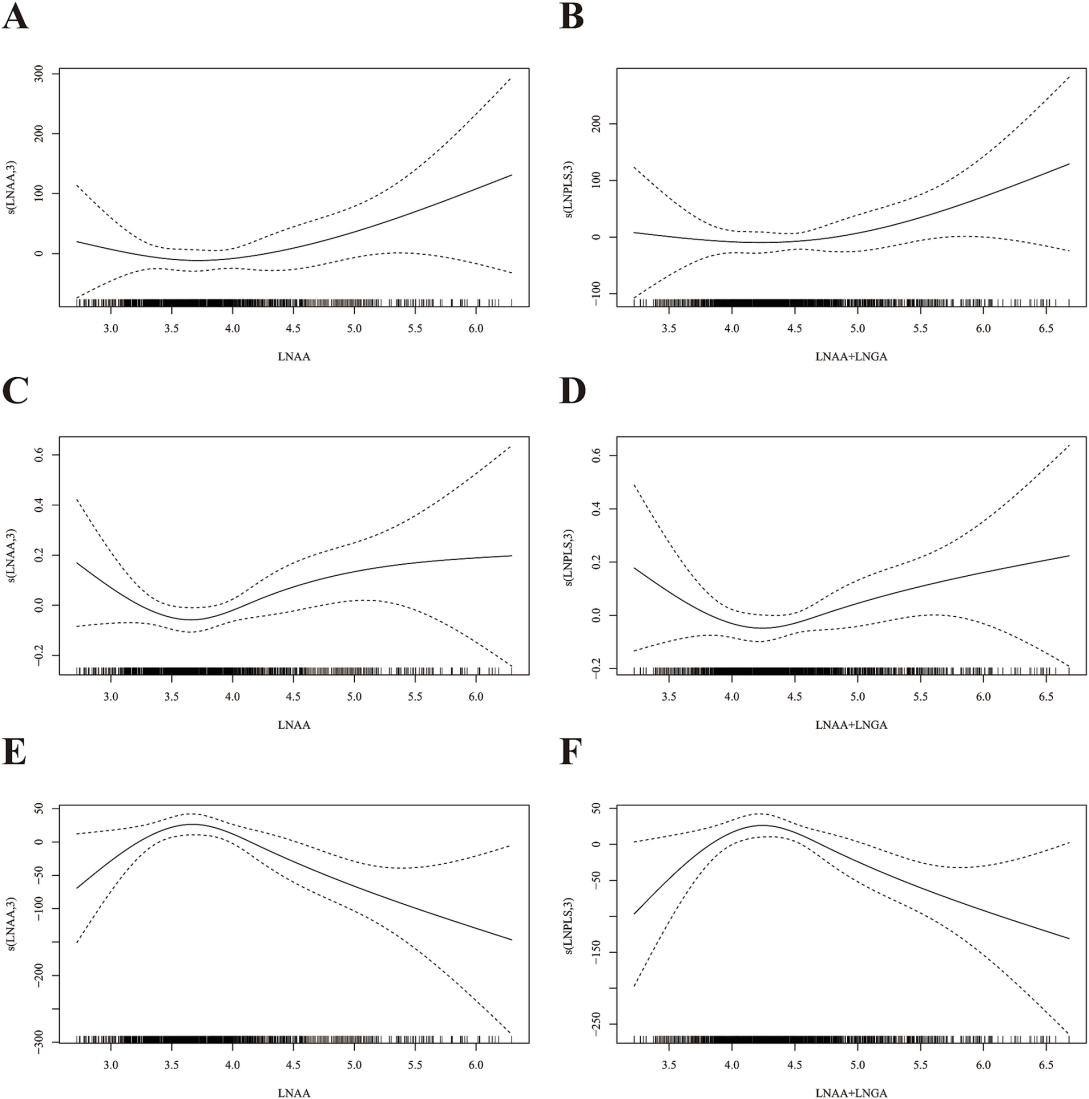
Dose–response relationships of AA hemoglobin biomarkers and markers of systemic inflammation and *α*-Klotho concentrations in serum. **(A)** HbAA and SII; **(B)** HbAA+HbGA and SII; **(C)** HbAA and SIRI; **(D)** HbAA+HbGA and SIRI; **(E)** HbAA and α-Klotho; **(F)** HbAA+HbGA and α-Klotho. LNAA, ln-transformed HbAA; LNAA+LNGA, ln-transformed HbAA+HbGA. The dose–response relationships were assessed by generalized additive models with adjustment for age, sex, race/ethnicity, educational level, body mass index, family poverty income ratio, cigarette smoking, alcohol consumption, and physical activity.

Nonlinear and linear associations between AA hemoglobin biomarkers and markers of systemic inflammation are displayed in Figure 2 and Table 2, respectively. No evidence of statistically significant nonlinear associations between AA hemoglobin biomarkers and systemic inflammation markers was found (*p*_nonlinearity_ > 0.05, Figure 2). HbAA, HbGA, and HbAA + HbGA were significantly positively correlated with SII and SIRI in the crude models (Model 1) and basic adjusted models (Model 2). No statistically significant association between AA hemoglobin biomarkers and SII or SIRI was observed after adjusting for potential confounders in Model 3.

**Table 2:** Estimated regression coefficients and 95% CI for markers of systemic inflammation and AA hemoglobin biomarkers.

	Model 1		Model 1		Model 1	
	β (95% CI)	*P*	β (95% CI)	*P*	β (95% CI)	*P*
SII						
HbAA	32.94 (5.05, 60.84)	0.021	32.16 (3.59, 60.73)	0.028	23.48 (−8.12, 55.08)	0.145
HbGA	47.43 (17.53, 77.34)	0.002	36.37 (5.59, 67.15)	0.021	24.88 (−7.85, 57.61)	0.136
HbAA+HbGA	42.17 (12.52, 71.82)	0.005	37.17 (6.79, 67.55)	0.017	26.84 (−6.29, 59.97)	0.112
HbGA/HbAA	27.42 (−23.63, 78.47)	0.293	−2.92 (−56.32, 50.48)	0.915	−0.97 (−58.63, 56.69)	0.974
SIRI						
HbAA	0.11 (0.03, 0.18)	0.008	0.08 (0.01, 0.16)	0.036	0.07 (−0.02, 0.15)	0.133
HbGA	0.10 (0.01, 0.18)	0.023	0.08 (−0.01, 0.16)	0.075	0.03 (−0.05, 0.12)	0.456
HbAA+HbGA	0.11 (0.03, 0.20)	0.008	0.09 (0.01, 0.17)	0.034	0.06 (−0.03, 0.15)	0.188
HbGA/HbAA	−0.07 (−0.21, 0.07)	0.342	−0.06 (−0.21, 0.08)	0.414	−0.11 (−0.27, 0.04)	0.154

β, regression coefficient; CI, confidence interval; SII, systemic immune-inflammation index; SIRI, system inflammation response index. AA biomarker data was ln-transformed. Model 1 was unadjusted. Model 2 was adjusted for age, sex, race/ethnicity. Model 3 was adjusted for age, sex, race/ethnicity, educational level, body mass index, family poverty income ratio, cigarette smoking, alcohol consumption, physical activity.

Table 3 illustrates the associations of AA hemoglobin biomarkers with serum concentrations of *α*-klotho. Higher HbAA concentration in whole blood was statistically significantly related to decreased serum concentrations of *α*-klotho (*β* = −35.76 pg./mL, 95% CI: −63.27, −8.25; *p* = 0.011), after adjustment for potential confounders. A negative association between HbAA + HbGA and serum concentrations of *α*-klotho was also observed (*β* = −33.82 pg./mL, 95% CI: −62.68, −4.96; *p* = 0.022).

**Table 3:** Estimated regression coefficients and 95% CI for serum α-Klotho concentrations and AA hemoglobin biomarkers.

	Model 1		Model 2		Model 3	
	β (95% CI)	*P*	β (95% CI)	*P*	β (95% CI)	*P*
HbAA	−31.54 (−55.93, −7.14)	0.011	−30.73 (−55.60, −5.85)	0.016	−35.76 (−63.27, −8.25)	0.011
HbGA	−18.22 (−44.45, 8.00)	0.173	−21.92 (−48.75, 4.92)	0.110	−24.98 (−53.51, 3.55)	0.086
HbAA+HbGA	−28.63 (−54.59, −2.67)	0.031	−29.64 (−56.12, −3.17)	0.028	−33.82 (−62.68, −4.96)	0.022
HbGA/HbAA	52.52 (7.92, 97.11)	0.021	41.27 (−5.20, 87.73)	0.082	41.50 (−8.73, 91.73)	0.106

β, regression coefficient; CI, confidence interval. AA biomarker data was ln-transformed. Model 1 was unadjusted. Model 2 was adjusted for age, sex, race/ethnicity. Model 3 was adjusted for age, sex, race/ethnicity, educational level, body mass index, family poverty income ratio, cigarette smoking, alcohol consumption, physical activity.

**Figure 3:**
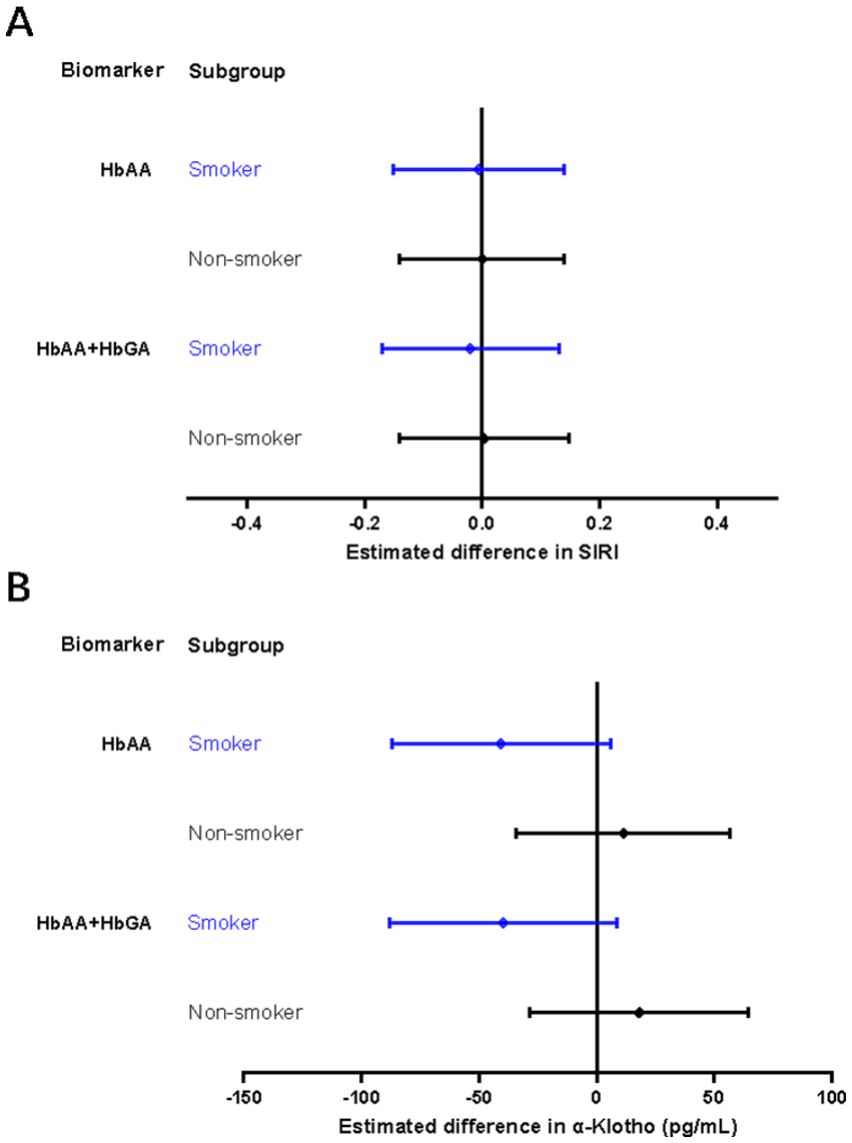
Stratified analysis by cigarette smoking for associations of AA hemoglobin biomarkers with SIRI **(A)** or serum α-Klotho concentrations **(B)**. SIRI, system inflammation response index. Models for smokers were adjusted for sex, age, race/ethnicity, educational level, family poverty-income ratio, body mass index, average cigarettes per day during past 30 days, alcohol consumption and physical activity. Models for non-smokers were adjusted for sex, age, race/ethnicity, educational level, family poverty-income ratio, body mass index, alcohol consumption, and physical activity.

The interaction term based on the multiplication of cigarette smoking and AA hemoglobin biomarkers was statistically significant in the associations between HbAA/HbGA and SII, as well as between HbAA+HbGA and α-klotho (Supplementary Table S1). After stratification by cigarette smoking, HbAA and HbAA + HbGA were borderline significantly associated with decreased serum concentrations of *α*-klotho only in smokers (β = −40.69 pg./mL, 95% CI: −87.15, 5.77; *p* = 0.086; β = −39.88 pg./mL, 95% CI: −88.23, 8.47; *p* = 0.106, respectively). No statistically significant association was observed with other AA hemoglobin biomarkers in the stratified analyses (Figure 3).

## 4 Discussion

This cross-sectional analysis revealed negative associations of HbAA and HbAA + HbGA with serum concentrations of α-klotho; the associations were more pronounced in smokers. HbAA and HbAA + HbGA showed no association with SII and SIRI, as calculated using blood cell counts.

Several epidemiological studies have addressed the association of AA exposure at environmentally relevant doses with systemic inflammation measured using various indicators. Our findings were consistent with those of previous studies (10, 26–28). In a pilot study, 14 healthy volunteers (6 smokers) were administered 160 g/day AA-containing potato chips (27). After 4 weeks, the concentrations of inflammation markers, plasma high-sensitivity CRP, high-sensitivity IL-6, gamma-glutamyltransferase significantly increased (*p* < 0.10) compared with baseline (before consumption) among smokers and nonsmokers (27). The urinary AA biomarkers in a Chinese population were significantly associated with increased concentrations of systemic inflammatory marker plasma CRP (28). This increase in plasma CRP concentration mediated 6.34–11.1% of the associations of urinary AA biomarkers with reduced pulmonary function. In a prospective study, Wang et al. (10) reported an association of the urinary AA biomarkers with 10-year cardiovascular disease risks in general adults, mediated by systemic inflammation (plasma CRP and circulating MPV), oxidative stress, and plasma transforming growth factor-β1. In NHANES 2003–2014 cycles, AA hemoglobin biomarkers were related to an increase in cancer mortality (mediated by low-grade INFLA-score), an inflammatory marker derived from CRP, white blood cell and platelet counts, and granulocyte/lymphocyte ratio (26). On the contrary, the present study indicated that AA exposure might not increase systemic inflammation in general adults. The reasons for inconsistent findings may be due to the heterogeneity between populations, outcome measurements, and time window of assessment. We cannot be ruled out that manifestations of inflammatory effects of AA may be temporarily masked by compensatory processes in this population and maybe become apparent in other study population. Moreover, CRP used in the previous studies may be a more sensitive biomarker in measuring inflammation, compared with SII and SIRI (21). Furthermore, given the short half-life of HbAA in humans, there may be exposure misclassification and the relevant time window for AA exposure and inflammation may not overlap.

Both *in vitro* and *in vivo* experimental studies have indicated that AA exposure increased inflammation in various tissues, including the neurons, brain, liver, and kidney (1). AA exposure induced an inflammatory response *in vitro* via the nuclear factor-κB (NF-κB) pathway in human PC12 cells (16). The transcription of inflammatory genes was enhanced after NF-κB activation, and pro-inflammatory cytokines, such as tumor necrosis factor-*α* (TNF-α), interleukin 6 (IL-6), pro-IL-1β, and pro-IL-18, were released. These findings were further observed in rodent models. AA treatment enhanced the serum concentrations of cytokines, including TNF-*α*, pro-IL-1β, and IL-6 (15, 34).

Several epidemiological studies have reported that reductions in serum α-Klotho levels were associated with several environmental contaminants, including heavy metals (35), perfluoroalkyl and polyfluoroalkyl substances (36), and polycyclic aromatic hydrocarbons (37). The exact biological mechanisms of action of AA exposure on the reduction in serum concentrations of *α*-klotho are still unclear. The most possible underlying mechanism was AA-induced oxidative stress. Several animal and epidemiological studies showed that AA exposure increases the levels of oxidative stress markers, such as urinary 8-hydroxydeoxyguanosine and 8-iso-prostaglandin-F2*α* (10, 15). The α-klotho stimulation upregulated the expression of phosphorylation forkhead box protein O3a, inhibiting ROS-related oxidative stress damage (24). ROS production and oxidative stress damage were negatively correlated with serum concentrations of *α*-klotho (25).

Stratified analyses revealed more prominent associations between AA exposure and serum concentrations of α-klotho in smokers than nonsmokers. This was also observed previously in associations of AA exposure with other health-related outcomes, including diabetes (12), cardiovascular diseases (38, 39), depressive symptoms (13). Cigarette smoking, a critical source of AA exposure, was associated with increases in local and systemic inflammation (40) and a reduction in serum concentrations of *α*-klotho (3, 41). Smokers had higher exposure levels of AA and other toxic chemicals, such as tar, formaldehyde, polycyclic aromatic hydrocarbons, and heavy metals (40), compared with nonsmokers. We cannot exclude the possibility that AA in combination with a series of toxic chemicals in tobacco smoke contributes to the decrease in serum concentrations of *α*-klotho in smokers. Residual confounding by smoking may also play a role in association between AA exposure and serum *α*-klotho concentrations. More epidemiological studies should be conducted to assess the exposure to a mixture of toxic chemicals associated with systemic inflammation and biological aging among active smokers.

This epidemiological study was novel in exploring the associations of internal AA exposure with novel systemic inflammation markers and serum concentrations of α-klotho in the general population. However, this study had several limitations. First, causality could not be inferred between AA exposure and systemic inflammation or serum concentrations of α-klotho owing to the observational study design, especially for the cross-sectional study design. Second, despite adjusting for a broad set of covariables, we could not exclude the possibility of residual confounders, such as occupational factors (42), and other environmental contaminants (35, 36). These may have confounding effects on the exposure-outcome associations. Third, HbAA biomarkers were assessed only once and thus reflect AA exposure over a short time window (42). This single measurement may lead to exposure misclassification, as the relevant time window for AA exposure and inflammation may not overlap. Repeated measurements would have provided a more accurate or long-term assessment of exposure levels.

## 5 Conclusion

AA exposure assessed using hemoglobin biomarkers was associated with decreased serum concentrations of *α*-klotho in general adults aged 40–79 years. The findings of this study provide suggestive evidence regarding the potential health effects of AA exposure at environmentally relevant doses. Future studies are warranted to identify potential biological mechanisms and develop intervention strategies.

## Data Availability

The raw data supporting the conclusions of this article will be made available by the authors, without undue reservation.

## Footnotes

^1^ https://www.r-project.org/
