# Supplementary material for: Association of acrylamide exposure with markers of systemic inflammation and serum alpha-klotho concentrations in middle-late adulthood
Source: Front Public Health. 2025 Mar 19;13:1457630. doi: 10.3389/fpubh.2025.1457630 (PMC11961959; doi:10.3389/fpubh.2025.1457630)
Supplement: Supplementary file 1 [file Table_1.DOCX]

***Supplementary Material***

**Contents**

[Table S1. Estimated regression coefficients and 95% CI for markers of systemic inflammation and AA hemoglobin biomarkers (*p* for interaction term). 2](#_Toc186465010)

# Table S1. Estimated regression coefficients and 95% CI for markers of systemic inflammation and AA hemoglobin biomarkers (interaction between AA and smoking).

|  | **SII** | | **SIRI** | | **α-Klotho** | |
| --- | --- | --- | --- | --- | --- | --- |
|  | β(95%CI) | *P-inter* | β(95%CI) | *P-inter* | β(95%CI) | *P-inter* |
| HbAA | 23.85 (-46.55, 94.25) | 0.507 | 0.06 (-0.13, 0.25) | 0.563 | -51.26 (-112.50, 9.99) | 0.101 |
| HbGA | -9.13 (-77.48, 59.22) | 0.794 | 0.03 (-0.15, 0.22) | 0.717 | -44.89 (-104.43, 14.65) | 0.140 |
| HbAA+HbGA | 6.17 (-66.73, 79.06) | 0.868 | 0.05 (-0.15, 0.25) | 0.630 | -54.30 (-117.74, 9.14) | 0.094 |
| HbAA/HbGA | -94.89 (-202.75, 12.97) | 0.085 | -0.12 (-0.41, 0.17) | 0.430 | 42.41 (-51.61, 136.44) | 0.377 |

**Abbreviations**: β- regression coefficient; CI-confidence interval; SII-systemic immune-inflammation index; SIRI: system inflammation response index

AA biomarker data was ln-transformed.

Models were adjusted for age, sex, race/ethnicity, educational level, body mass index, family poverty income ratio, cigarette smoking, alcohol consumption, physical activity and AA biomarker * cigarette smoking
